# DNA-programmed cell assembly: from cells, tissues to organoids

**DOI:** 10.3389/fbioe.2025.1716071

**Published:** 2025-10-28

**Authors:** Zhenyi Chen, Pan Fu, Kaizhe Wang

**Affiliations:** ^1^ School of Materials Science and Chemical Engineering, Ningbo University, Ningbo, China; ^2^ Ningbo Key Laboratory of Biomedical Imaging Probe Materials and Technology, Ningbo Cixi Institute of Biomedical Engineering, Ningbo Institute of Materials Technology and Engineering, Chinese Academy of Sciences, Ningbo, China

**Keywords:** cell assembly, DNA nanomaterials, cell engineering, cell-cell interactions, tissue models, organoids

## Abstract

The precise spatial organization of cells into functional tissues represents a fundamental challenge in biology and regenerative medicine. Conventional methods for directing cell assembly often lack the specificity, reproducibility, and dynamic control necessary to mimic native tissue architectures. This review explores the emerging use of DNA as a programmable and biocompatible strategy to engineer cell–cell interactions and construct hierarchically ordered tissue models. We first introduce the properties of various DNA toolbox and their strategies for cell modification and assembly. Importantly, we highlight the latest research advances in DNA-encoded cell spheroids, layered tissues, and organoids. Finally, we summarize current challenges and future directions in DNA-programmed assembly.

## 1 Introduction

Morphogenesis in living systems is a complex and precise process driven by the spatiotemporally programmed self-assembly of cells into hierarchical architectures with defined structural and functional features (Lancaster and Knoblich, 2014). The morphology of tissues and organs, such as the chambered design of the heart, the lobular arrangement of the liver, and the intricate circuitry of neurons, is not merely a product of physical accumulation but reflects the ordered integration of specialized functional units (Zaret, 2002; Lancaster et al., 2013; Hofbauer et al., 2021). The ability to reconstruct such architectures *in vitro*, including multicellular clusters, tissues, and organoids, offers powerful tools for elucidating developmental principles and advancing applications in disease modeling, tissue regeneration, and drug screening (Escopete et al., 2025; Kim et al., 2025; Li M. Q. et al., 2025). Conventional tissue engineering techniques, such as hanging-drop (Wang S. H. et al., 2017), liquid-overlay (Martinez et al., 2015), spinner flasks and bioreactors (Jo et al., 2018), microfluidic devices (Park et al., 2019), and magnetic levitation (Jaganathan et al., 2014), enable the formation of cellular spheroids but often lack precision. These methods typically yield structures with heterogeneous size, composition, and poor reproducibility due to stochastic cell placement, thereby limiting their biomimetic fidelity and functionality (Lancaster et al., 2017; Hofer and Lutolf, 2021). Thus, a central challenge in the field is the engineering of robust and programmable control over cell–cell recognition and spatial organization.

DNA as a biomolecule renowned for its dual role in genetic encoding and precise molecular self-assembly, offers a unique platform for nanoscale engineering (Hu et al., 2019). Through Watson–Crick base pairing, DNA enables predictable and programmable construction of nanostructures with high fidelity (Sun et al., 2018; Xia et al., 2021; Liu et al., 2023). It can emulate natural ligand–receptor recognition mechanisms and mediate the assembly of diverse natural and synthetic materials across nano-to millimeter scales (He et al., 2008; Gu et al., 2010). DNA-programmed assembly of cells (DPAC) involves functionalizing cell membranes with DNA-based nanodevices, enabling selective recognition between cells bearing complementary sequences and facilitating the programmable construction of complex tissue mimics (Lin et al., 2022; Chen et al., 2024; Li Q. et al., 2025). By tuning the length, sequence, and structural configuration of DNA, as well as its surface density on cells, DNA can precisely control the strength, specificity, and logic-gated dynamics of intercellular adhesion. Moreover, strand displacement and other dynamic DNA reactions allow reversible, real-time switching of cell–cell binding, mirroring developmental processes (Xu W. W. et al., 2025). Beyond adhesion, DNA modules can act as signal transducers, environmental sensors, and drug delivery vehicles, enabling synergistic programming of structure and function (Shen et al., 2024).

This review comprehensively discusses strategies for programming cellular assembly using DNA materials. First, we elucidate DNA functional toolboxes, methods for cell surface modification, and assembly strategies. We then highlight applications of DNA materials in constructing multicellular architectures across varying scales and in regulating tissue-level functions. Finally, we discuss current challenges and future prospects in the field.

## 2 Strategies for DNA-encoded cell assembly

### 2.1 DNA toolbox

DNA self-assembly furnishes a versatile toolbox for cellular manipulation from the molecular to the mesoscale, including DNA duplex, DNA tetrahedra, DNA origami, and DNA hydrogels, thereby enabling multiscale and multifunctional control over cells.

**FIGURE 1:**
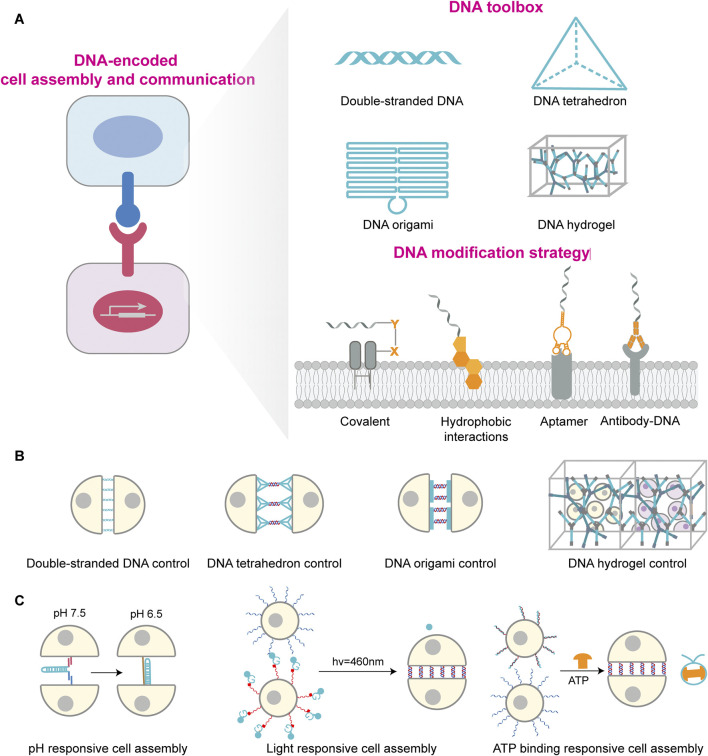
DNA-mediated cellular assembly and regulation: toolbox, modification strategies, and stimulus-responsive systems. **(A)** DNA-encoded cell assembly and communication schematic on the left. DNA toolbox library shown at top right, including double-stranded DNA, DNA tetrahedron, DNA origami and DNA hydrogel, which provides molecular regulatory tools for cellular interactions; The lower right side shows DNA modification strategies on cell membrane, including covalent linkage, hydrophobic insertion, aptamer binding and antibody recognition, to achieve stable anchoring of DNA molecules on cell membrane surface. **(B)** Different DNA structural units regulate cellular assembly patterns, including double-stranded DNA, DNA tetrahedron, DNA origami, and DNA hydrogel-regulated cellular assembly mechanisms. **(C)** Stimulus-responsive cellular assembly systems comprise pH, light, and ATP response units. These systems induce dynamic conformational changes or interactions in DNA through external stimuli, enabling controlled regulation and reversible switching of cellular assembly.

DNA duplex comprises two complementary strands stabilized by hydrogen bonding between specific base pairs and provides a flexible and controllable platform for programmable cell assembly (Figure 1A) (Hoffecker et al., 2019; Louis et al., 2022). This approach is simple and adaptable, as binding strength and specificity can be tuned by altering hybridization length and sequence. For example, Gartner et al. employed single-stranded DNA (ssDNA) modifications on cell membranes, wherein complementary ssDNA sequences attached to another cell’s membrane. Upon cell proximity, complementary sequences hybridised, forming stable connections via a “lock-and-key” mechanism that prevented cell drift (Figure 1B) (Gartner and Bertozzi, 2009). However, DNA duplex exhibits relatively poor stability due to nuclease degradation and finite binding strength. To enhance durability, strategies such as chemical backbone modifications and terminal protection have been employed (Kwak and Herrmann, 2011). Furthermore, Shi et al. developed multivalent DNA molecules capable of binding multiple complementary sequences, thereby increasing the number of cells that can be assembled while reducing the required DNA density on each cell (Shi et al., 2018). Importantly, designing more rigid two-dimensional and three-dimensional DNA framework structures can reduce nuclease accessibility and enhance structural synergy, thereby improving stability (Lv et al., 2025; Wang et al., 2025).

DNA tetrahedron are rigid three-dimensional nanostructures assembled bottom-up from DNA strands (Figure 1A) (Goodman et al., 2005). Compared to DNA duplexes, tetrahedral DNA offer geometrical rigidity and enable precise spatial positioning of functional elements down to the nanometer scale, thus allowing fine control over intercellular assembly (Figure 1B) (Zhang et al., 2024). Xun et al. demonstrated that the DNA tetrahedron with three hydrophobic vertices stably to the plasma membrane for hours compared to ssDNA, providing a robust platform for cell–interface engineering (Li J. et al., 2019). Du et al. anchored DNA tetrahedron to antigen-presenting cells (APCs) and precisely tuned the intermembrane spacing between APCs and T cells. Reducing this spacing significantly enhanced T cell receptor triggering and activation by combining additional mechanical forces effect with strict CD45 exclusion, revealing a distance-dependent mechanism in immunological synapse formation (Du et al., 2023). Owing to the mechanical rigidity and geometric stability of DNA tetrahedron, Xiao et al. employed DNA tetrahedron to enhance the affinity of receptors binding to cancer cells. This approach also strengthened the anchoring stability of receptors on cell membranes, ultimately significantly promoting the interaction between NK cells and cancer cells and their killing efficiency (Xiao et al., 2022).

DNA origami, created by folding a long scaffold strand with hundreds of short staples, yields two- and three-dimensional structures with nanometer precision (Figure 1A) (Rothemund, 2006; Andersen et al., 2009). Relative to simple duplex-mediated recognition, origami upgrades “point-to-point” hybridization to “patterned” adhesion with defined spatial architectures, thereby improving the precision and topological complexity of cell assembly (Figure 1B) (Wagenbauer et al., 2023; Li et al., 2024). Akbari et al. used membrane-anchored DNA origami as a molecular-scale membrane-bound breadboard (MBB) to program both homotypic and heterotypic cell–cell binding, mimicking receptor–ligand-mediated recognition and signaling on biological membranes (Akbari et al., 2017). By controlling aptamer identity, valency, and spatial arrangement on origami, Hu et al. developed a series of adjustable multivalent aptamer-based DNA nanostructures. These structures not only discriminate tumor types and emulate multiheteroreceptor-mediated recognition but also guide specific interactions between macrophages and tumor cells, thereby leading to effective immune clearance. This demonstrates great potential for personalized tumor treatment (Hu et al., 2024).

DNA hydrogels are three-dimensional polymer networks formed by DNA hybridization, combine programmability and biocompatibility, serving as artificial extracellular matrices that provide mechanical support for cells (Figure 1A) (Um et al., 2006; Xing et al., 2011). Peng et al. designed a dynamic DNA cross-linked matrix that enables computational predictability and systematic regulation of its viscoelastic, thermodynamic, and kinetic parameters by modifying sequence information (Figure 1B) (Peng et al., 2024). These matrices support diverse cell types and guide polarization and morphogenesis by tuning adhesive ligands and stress relaxation, providing a programmable platform to model tissue mechanics and cell–matrix mechanobiology. Exploiting the tunable mechanics and photoresponsiveness of DNA hydrogels, Afting et al. fabricated nanoengineered DNA microspheres with tissue-mimetic, tunable stiffness. These enable spatiotemporally controlled release of morphogenetic factors within organoids, thereby inducing retinal organoids exhibiting in vivo-like cellular diversity and reproducing morphogen gradient-driven pattern formation processes (Afting et al., 2024).

### 2.2 Cell-surface DNA modification

Efficient, precise, and stable anchoring of DNA to the cell surface is foundational for cell-surface engineering and programmable assembly (Figure 1A). Direct chemical conjugation forms covalent bonds to lysine or cysteine residues on membrane proteins, including amine coupling, thiol coupling, and glycoengineering-based conjugation (Baskin et al., 2007; Stephan et al., 2010; Vermesh et al., 2011; Yang et al., 2015). These methods offer stability and broad applicability, but covalent protein modification can compromise modification efficiency and controllability, and complex operations may affect cell viability, and native function (Charter et al., 2002; Vogel et al., 2013; Shi and Wang, 2021). Hydrophobic insertion utilizes lipophilic groups such as cholesterol, tocopherol, or long aliphatic chains to embed into the lipid bilayer, thereby exposing DNA to the extracellular environment (You et al., 2017; Li H. Y. et al., 2019; Guo et al., 2022; Ma et al., 2024). This strategy is simple, general, minimally disruptive to membranes, and highly designable (Jin et al., 2019), but may suffer from probe aggregation and DNA internalization or shedding (Borisenko et al., 2009; Kwak and Herrmann, 2011; Selden et al., 2012). Antibody-mediated targeting exploits antibody–antigen recognition to localize DNA on specific cells with high specificity and low off-target effects, but is constrained by surface protein heterogeneity, antibody costs, conjugation complexity, and quality control requirements (Bailey et al., 2007; Saka et al., 2019; Dacon et al., 2022; He et al., 2023). Aptamer-based targeting offers flexible, customizable recognition at defined sites, is compatible with downstream DNA reactions, and is well-suited for specific recognition and dynamic control during cell assembly (Ni et al., 2017; Li L. et al., 2019; Yu et al., 2021; Yao et al., 2024).

### 2.3 DNA-encoded cell assembly

Once ssDNA, DNA tetrahedra, or DNA origami are stably anchored on the membrane, cells can be arranged and assembled with controlled geometries. DNA hydrogels provide scaffolding with spatial programmability and photoresponsiveness. Building on membrane functionalization, the programmable reactivity of DNA enables dynamic and intelligent modulation of intercellular interactions, opening routes to biomimetic communication and programmed assembly (Figure 1C) (Li L. X. et al., 2025). Hou et al. devised DNA triplex nanoswitches (DTNs) that incorporate Hoogsteen interactions, allowing conformational switching within physiological pH ranges while preserving Watson–Crick stability (Hou et al., 2021). Fluctuations in microenvironmental pH thus trigger controlled recognition and binding, enabling pH-dependent assembly, a useful feature for acidic niches such as the tumor microenvironment. Light-responsive DNA hybridization technology offers a difference regulatory approach by activating or deactivating DNA-binding capabilities through light exposure. As shown by Mathis et al., photocleavable protecting groups embedded in DNA strands can be removed under defined wavelengths (425–450 nm) to restore hybridization, thereby triggering rapid, spatiotemporally resolved cell–cell adhesion (Mathis et al., 2023). In addition, ATP-responsive DNA self-assembly provides an orthogonal route to dynamic multicellular regulation. Xu et al. harnessed antagonistic enzyme pairs, T4 DNA ligase and BamHI restriction endonuclease, to respond to physiological ATP concentrations and maintain dynamic steady states of DNA monomer assembly. Using ATP as a chemical fuel, the system affords reversible regulation of multicellular interactions, offering a means to emulate energy-dependent cellular behaviors in immunity and tissue regeneration (Xu Y. et al., 2025).

## 3 DNA encoding across organizational levels of cell assemblies

### 3.1 Cell clusters and spheroids

**FIGURE 2:**
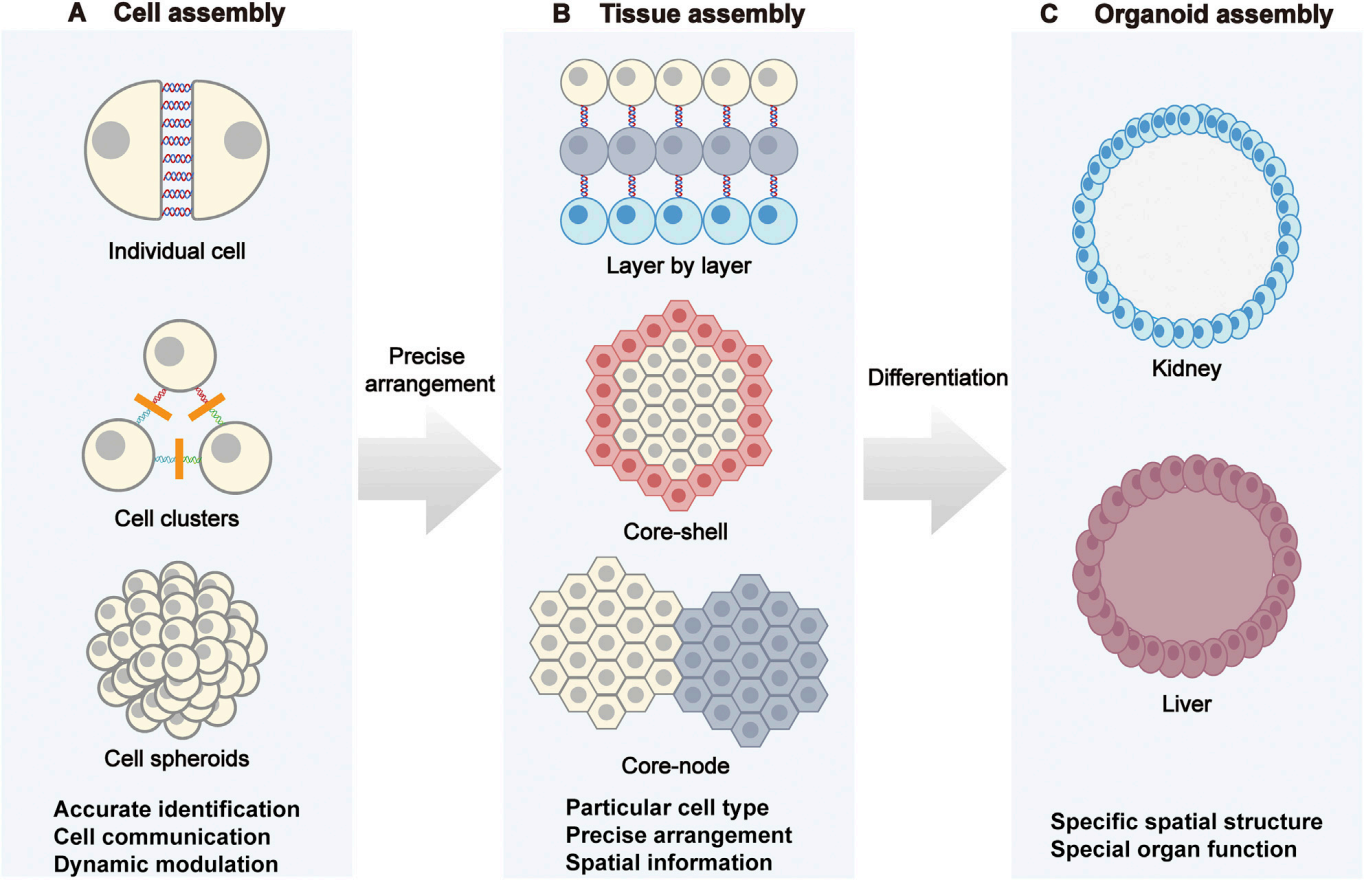
DNA-programmed assembly at the cellular, tissue, and organoid levels. **(A)** Cellular assembly: Through DNA-mediated processes, individual cells progressively form cell clusters and ultimately assemble into cell spheroids. **(B)** Tissue assembly: Strategies such as layer-by-layer stacking, core-shell structures, and core nodes are employed to program specific cell types, achieving precise cellular arrangement and endowing tissues with spatial information. **(C)** Organoid assembly: DNA programming regulates cell differentiation to construct organoids like kidneys and livers with specific spatial structures, recreating the distinct functions of natural organs.

Cell clusters and spheroids are fundamental units for probing structural stability, intercellular communication, and microenvironmental self-organization (Figure 2A) (Stevens et al., 2023). In DNA-programmed cellular assembly, the adhesion energy between cells depends on the hybridization free energy (ΔG) of DNA strands and their membrane anchoring density. Arora et al. found that when varying the length of base-pairing regions, molecular adhesion energies corresponding to 12 bp and 45 bp binding regions were 2.8 × 10^6^ kT and 10.7 × 10^6^ kT, respectively (Arora et al., 2025). Furthermore, anchoring density regulates the number of bonds that can form between adjacent cell surfaces. Gartner et al. achieved up to a 20-fold change in assembly rate by reducing the DNA chain concentration from 150 μM to 25 μM (Gartner and Bertozzi, 2009). Therefore, by regulating intercellular pairing and coupling, microaggregate formation, and spheroid assembly via DNA, the geometric structure and adhesion energy landscape of intercellular contacts can be established at an early stage, thereby enabling precise control of cellular functions (Chandra et al., 2006; Cui et al., 2022). DNA-encoded single-cell pairing employs the toolbox above to create highly selective heterotypic coupling. For example, Prahl et al. used augment DNA ‘‘velcro’’ technology for selective patterning of ssDNA labeled cells on mechanically defined photoactive polyacrylamide hydrogels, achieving high-precision spatial arrangement and long-term co-culture of multiple cell types and enabling studies of how epithelial–mesenchymal interface geometry influences cytoskeletal alignment and extracellular signal-related kinase (ERK) signaling, thereby modeling the interplay between mechanics and biochemistry at tissue boundaries (Prahl et al., 2023). Extending these capabilities with DNA framework nanostructures, Guo et al. realized reversible and tunable cell assembly and cargo transfer, underscoring the advantages of DNA nanostructures for dynamic control of cell–cell interactions (Guo et al., 2022). Wen et al. used amphiphilic aptamer-incorporated DNA tetrahedra that selectively pair T cells with monocytes, leveraging the intrinsic tumor-homing capability of monocytes to enhance intratumoral T cell infiltration, pointing to new avenues in immune engineering and cancer therapy (Wen et al., 2025).

Programmed DNA-mediated assembly can drive spontaneous integration of cell–cell and cell–matrix interactions to form spheroids and other higher-order aggregates. Compared with stochastic clustering via native adhesion, DNA coding confers high customizability over topology, layer ratios, and boundary shapes (Mathis et al., 2024). Liu et al. used DNA-encoded surface modification to program intercellular connections and controlled 3D tissue self-assembly, constructing functional signaling pathway that revealed the consequences of cell-to-cell variability in Ras activation on the morphogenesis of mammary epithelial cells, highlighting the utility of DNA programming for modeling tissue microenvironments and signal transduction (Liu et al., 2012). Such processes can be dynamically tuned by varying sequence complementarity and binding strength. Likewise, Kong et al. leveraged programmable base pairing to engineer a modular receptor system across diverse microbes, including Gram-positive bacteria, Gram-negative bacteria, and spores. This system enables the controlled assembly and dynamic response of microbial clusters, further expanding the application of DNA-programmed assembly in synthetic biology and microbial community engineering (Kong et al., 2025).

### 3.2 Layered tissue construction

Multiscale tissues exhibit horizontal layering, nested architectures, regionalization, and functional zoning that determine polarity, barrier function, mass transport, and system-level force transmission. DNA nanotechnology enables micrometer-scale reconfiguration of interfacial interactions to control multilayer assemblies with precision (Figure 2B) (Huang et al., 2024).

The assembly of spheroids can be directed by designing sequence complementarity and binding strength to control orientation and positioning (Li et al., 2015). In planar layered structure, Todhuner et al. employs sequence-specific strands as “Velcro” to arrange distinct cell types with single-cell precision on two-dimensional substrates, enabling programmed construction of three-dimensional microtissues with defined spatial heterogeneity (Todhunter et al., 2015). Furthermore, Viola et al. developed a “kinomorphs” based on photolithographic DPAC (pDPAC), embedding fibroblasts as “crease blocks” in extracellular matrix to guide cell–cell connectivity. Resulting traction forces drive morphogenetic transformations that culminate in cell cluster fusion and lumen formation at prescribed locations, demonstrating strong potential for constructing polarized, functionally luminal tissues for regenerative medicine and disease modeling (Viola et al., 2020).

For core–shell architectures, DNA nanotechnology affords programmable spatial organization that emulates the regionalized distributions of cells in native tissues. Decorating cells with defined DNA sequences as synthetic receptors enables type-specific recognition and adhesion based on complementarity, guiding the formation of stable core–shell units (Gao et al., 2019). Li et al. introduced the expansion of stem cells with pairing niches (ESPN) strategy, anchoring cholesterol-modified ssDNA to cell membranes to achieve pairing between mammary stem cells and niche cells, which promoted stem cell proliferation and maintenance (Li et al., 2018). Using photolithography combined with DNA hybridization, Scheideler et al. patterned multiple cell types and ligands with micron-level precision to build multilayer constructs that integrate malignant breast epithelial cells, nonmalignant breast epithelial cells, and endothelial cells to model complex physiological systems such as the tumor microenvironment (Scheideler et al., 2020). This approach offers high precision and flexibility and is well-suited for dissecting intercellular signaling, tissue development, and disease mechanisms.

DNA nanotechnology has also been applied in constructing dynamic core-node architectures, which are suitable for simulating spatial reorganization and signal transduction occurring during developmental or pathological processes (Kong et al., 2025). Wang et al. proposed a “brick-to-wall” strategy that exploits the self-healing and biocompatibility of DNA supramolecular hydrogels to encapsulate different cells in discrete modules that fuse into multicellular core–node constructs supporting migration and interaction in 3D (Wang Y. J. et al., 2017). Qian et al. developed DNAzyme-based ion-responsive systems that use Zn^2+^ or Mg^2+^ to trigger controlled disassembly and reassembly of clusters, even enabling reversible connections and directed migration among spheroids. Such methods support logical operations, including AND/OR gating, and provide molecular-level control tools for intelligent tissue models, dynamic drug screening platforms, and reconfigurable biological systems (Qian et al., 2021).

### 3.3 Organoid construction

Organoids emulate organ architectures and functions by precisely controlling cell positioning and interactions, providing reliable models for disease modelling, drug screening, and regenerative medicine (Figure 2C) (Rookmaaker et al., 2015; Pasca et al., 2025). The sequence-specific programmability of DNA enables precise control of adhesion, spatial localization, and cell ratios, addressing bottlenecks in structural integrity, functional maturation, and batch-to-batch consistency in conventional organoid culture. DNA nanotechnology offers powerful means to realize highly biomimetic and controllable organoids by coupling molecular recognition with programmable assembly, thereby mitigating variability and structural or functional deficits. By tuning cell–cell adhesion, matrix mechanics, and multicellular composition, DNA-guided processes steer organoid formation in space and time to model organ development and pathology (Arora et al., 2025; Zhu et al., 2025). Weber et al. exploited reversible adhesion via membrane-anchored ssDNA to create a “Chemical Micromolding” approach for rapid, controlled 3D aggregation of multiple cell types. Supported in Matrigel, this method reproduced the spatial morphologies of natural tissues such as mammary glands while preserving self-organization and microenvironmental adaptability (Weber et al., 2016). To reduce variability associated with Matrigel, Porter et al. applied pDPAC with DNA “velcro” in microwell arrays to precisely control ratios of nephron progenitors and ureteric bud cells, achieving structurally consistent kidney organoids in 97% of wells and markedly improving proximal tubule structures morphogenesis over conventional methods (Porter et al., 2024). To enhance uniformity and functional maintenance, Wei et al. demonstrates the superior efficiency and reproducibility offered by DNA origami technology. Specifically, the resulting spheroids exhibited remarkably low size variability (<3.7%) and could be reliably generated within 24 h, in contrast to the rough and heterogeneous structures formed by the conventional hanging-drop method. Moreover, this approach bypasses the cell number limitations of the liquid-overlay technique, efficiently producing spheroids across a wide range of cellularities (50–1,000,000 cells) (Wei et al., 2024). This high degree of control over the assembly process ensures consistent outcomes, highlighting the enhanced reproducibility of DNA-programmed assembly (Zhu et al., 2024; Luo et al., 2025). Collectively, these representative studies illustrate the breadth and control afforded by DNA nanotechnology for building high-fidelity organoids, enabling precise and reliable platforms for disease modeling, pharmacology, and regenerative applications.

## 4 Challenges and outlook

DNA-encoded cell assembly offers novel engineering strategies for constructing complex tissues and organoids. Its advantage lies in achieving programmable control over cell types, numbers, relative positions, and functions through sequence information. However, this field still faces multiple challenges. First, DNA currently serves primarily as a temporary structural linker during the initial assembly phase. Exposed DNA rapidly degrades, leaving subsequent development of cellular assemblies largely dependent on spontaneous cellular interactions (Wang et al., 2023; Yao et al., 2024). There is an urgent need to develop novel material strategies enabling DNA materials to provide sustained regulation of structure and function throughout the entire lifecycle of the assembly. Second, existing DNA-encoded assembly techniques provide insufficient control of the microenvironment and struggle to recapitulate the complex *in vivo* gradients of biochemical and biophysical cues, resulting in gaps in morphology and functional maturation (Zhu et al., 2024; Yamada et al., 2025). Third, dense DNA modifications may potentially interfere with the function of native signaling receptors or adhesion molecules on the cell membrane. By optimizing DNA modification density, employing spatially controllable DNA nanomaterials for targeted modification to avoid random coverage of critical receptor regions, and developing stimulus-responsive DNA systems, precise cellular programming can be achieved while maximally preserving the cell’s native biological functions. Finally, current approaches largely achieve simple co-assembly of different cell types, but lack precise, higher-order structural control and coupling across functional hierarchies, such as vascularization, innervation, and immune infiltration (Dao et al., 2024; Kim et al., 2024; Lo et al., 2025).

Future advances in DNA-programmed cell assembly will likely emerge from integration with multidisciplinary technologies, bridging fundamental research and clinical translation. For example, coupling with 3D bioprinting could use DNA-encoded cellular modules as “bioinks” for high-precision spatial stacking to rapidly build macroscale, architecturally complex organ structures, while coordinating graded mechanics and porosity during printing to establish ordered biomechanical microenvironments. Integrating patient-derived induced pluripotent stem cells (iPSCs) enables assembly of DNA-encoded cells and organoids into personalized, multilayered constructs with specified tissue functions. Incorporating machine learning and artificial intelligence can elucidate and optimize the mapping between DNA sequence–structure and construct function, accelerating the development of intelligently responsive tissue materials. Such convergence will propel DNA-programmed assembly from static construction to dynamic regulation and functional integration, enabling the on-demand fabrication of physiologically functional tissues and organs.

## References

[B1] Afting C. Walther T. Drozdowski O. M. Schlagheck C. Schwarz U. S. Wittbrodt J. (2024). DNA microbeads for spatio-temporally controlled morphogen release within organoids. Nat. Nanotechnol. 19 (12), 1849–1857. 10.1038/s41565-024-01779-y 39251862 PMC11638066

[B2] Akbari E. Mollica M. Y. Lucas C. R. Bushman S. M. Patton R. A. Shahhosseini M. (2017). Engineering cell surface function with DNA origami. Adv. Mat. 29 (46), 1703632. 10.1002/adma.201703632 29027713 PMC5739518

[B3] Andersen E. S. Dong M. Nielsen M. M. Jahn K. Subramani R. Mamdouh W. (2009). Self-assembly of a nanoscale DNA box with a controllable lid. Nature 459 (7243), 73–76. 10.1038/nature07971 19424153

[B4] Arora A. Rizvi M. S. Grenci G. Dilasser F. Fu C. Y. Ganguly M. (2025). Viscous dissipation in the rupture of cell-cell contacts. Nat. Mat. 24 (7), 1126–1136. 10.1038/s41563-025-02232-8 40355570

[B5] Bailey R. C. Kwong G. A. Radu C. G. Witte O. N. Heath J. R. (2007). DNA-Encoded antibody libraries: a unified platform for multiplexed cell sorting and detection of genes and proteins. J. Am. Chem. Soc. 129 (7), 1959–1967. 10.1021/ja065930i 17260987 PMC3677962

[B6] Baskin J. M. Prescher J. A. Laughlin S. T. Agard N. J. Chang P. V. Miller I. A. (2007). Copper-free click chemistry for dynamic *in vivo* imaging. Proc. Natl. Acad. Sci. U.S.A. 104 (43), 16793–16797. 10.1073/pnas.0707090104 17942682 PMC2040404

[B7] Borisenko G. G. Zaitseva M. A. Chuvilin A. N. Pozmogova G. E. (2009). DNA modification of live cell surface. Nucleic Acids Res. 37 (4), e28. 10.1093/nar/gkn1034 19158188 PMC2651772

[B8] Chandra R. A. Douglas E. S. Mathies R. A. Bertozzi C. R. Francis M. B. (2006). Programmable cell adhesion encoded by DNA hybridization. Angew. Chem. Int. Ed. 45 (6), 896–901. 10.1002/anie.200502421 16370010

[B9] Charter N. W. Mahal L. K. Koshland D. E. Bertozzi C. R. (2002). Differential effects of unnatural sialic acids on the polysialylation of the neural cell adhesion molecule and neuronal behavior. J. Biol. Chem. 277 (11), 9255–9261. 10.1074/jbc.M111619200 11786551

[B10] Chen H. Ding Q. Li L. Wei P. Niu Z. Kong T. (2024). Extracellular vesicle spherical nucleic acids. JACS Au 4 (6), 2381–2392. 10.1021/jacsau.4c00338 38938802 PMC11200237

[B11] Cui H. C. Zhang T. Q. Kong Y. H. Xing H. Wei B. (2022). Controllable assembly of synthetic constructs with programmable ternary DNA interaction. Nucleic Acids Res. 50 (12), 7188–7196. 10.1093/nar/gkac478 35713533 PMC9262601

[B12] Dacon C. Tucker C. Peng L. H. Lee C. C. D. Lin T. H. Yuan M. (2022). Broadly neutralizing antibodies target the coronavirus fusion peptide. Science 377 (6607), 728–735. 10.1126/science.abq3773 35857439 PMC9348754

[B13] Dao L. You Z. Lu L. Xu T. Y. Sarkar A. K. Zhu H. (2024). Modeling blood-brain barrier formation and cerebral cavernous malformations in human PSC-derived organoids. Cell. Stem Cell. 31 (6), 818–833.e11. 10.1016/j.stem.2024.04.019 38754427 PMC11162335

[B14] Du Y. L. Lyu Y. Lin J. Ma C. R. Zhang Q. Zhang Y. T. (2023). Membrane-anchored DNA nanojunctions enable closer antigen-presenting cell-T-cell contact in elevated T-cell receptor triggering. Nat. Nanotechnol. 18 (7), 818–827. 10.1038/s41565-023-01333-2 36894782

[B15] Escopete S. Arzt M. Mozneb M. Moses J. Sharma A. (2025). Human cardiac organoids for disease modeling and drug discovery. Trends Mol. Med. 10.1016/j.molmed.2025.08.004 40914706

[B16] Gao T. Chen T. S. Feng C. He X. Mu C. L. Anzai J. (2019). Design and fabrication of flexible DNA polymer cocoons to encapsulate live cells. Nat. Commun. 10, 2946. 10.1038/s41467-019-10845-2 31270421 PMC6610073

[B17] Gartner Z. J. Bertozzi C. R. (2009). Programmed assembly of 3-dimensional microtissues with defined cellular connectivity. Proc. Natl. Acad. Sci. U.S.A. 106 (12), 4606–4610. 10.1073/pnas.0900717106 19273855 PMC2660766

[B18] Goodman R. P. Schaap I. A. T. Tardin C. F. Erben C. M. Berry R. M. Schmidt C. F. (2005). Rapid chiral assembly of rigid DNA building blocks for molecular nanofabrication. Science 310 (5754), 1661–1665. 10.1126/science.1120367 16339440

[B19] Gu H. Z. Chao J. Xiao S. J. Seeman N. C. (2010). A proximity-based programmable DNA nanoscale assembly line. Nature 465 (7295), 202–205. 10.1038/nature09026 20463734 PMC2872101

[B20] Guo Z. Z. Zhang L. L. Yang Q. X. Peng R. Z. Yuan X. Xu L. J. (2022). Manipulation of multiple cell-cell interactions by tunable DNA scaffold networks. Angew. Chem. Int. Ed. 61 (7), e202111151. 10.1002/anie.202111151 34873818

[B21] He Y. Ye T. Su M. Zhang C. Ribbe A. E. Jiang W. (2008). Hierarchical self-assembly of DNA into symmetric supramolecular polyhedra. Nature 452 (7184), 198–201. 10.1038/nature06597 18337818

[B22] He Y. B. Ge C. R. Moreno-Giró A. Xu B. Z. Beusch C. M. Sandor K. (2023). A subset of antibodies targeting citrullinated proteins confers protection from rheumatoid arthritis. Nat. Commun. 14 (1), 691. 10.1038/s41467-023-36257-x 36754962 PMC9908943

[B23] Hofbauer P. Jahnel S. M. Papai N. Giesshammer M. Deyett A. Schmidt C. (2021). Cardioids reveal self-organizing principles of human cardiogenesis. Cell. 184 (12), 3299–3317.e22. 10.1016/j.cell.2021.04.034 34019794

[B24] Hofer M. Lutolf M. P. (2021). Engineering organoids. Nat. Rev. Mat. 6 (5), 402–420. 10.1038/s41578-021-00279-y 33623712 PMC7893133

[B25] Hoffecker I. T. Arima Y. Iwata H. (2019). Tuning intercellular adhesion with membrane-anchored oligonucleotides. J. R. Soc. Interface. 16 (159), 20190299. 10.1098/rsif.2019.0299 31662069 PMC6833338

[B26] Hou J. J. Zhu S. T. Zhao Z. W. Shen J. L. Chao J. Shi J. Y. (2021). Programming cell communications with pH-responsive DNA nanodevices. Chem. Commun. 57 (37), 4536–4539. 10.1039/d1cc00875g 33956003

[B27] Hu Q. Q. Li H. Wang L. H. Gu H. Z. Fan C. H. (2019). DNA nanotechnology-enabled drug delivery systems. Chem. Rev. 119 (10), 6459–6506. 10.1021/acs.chemrev.7b00663 29465222

[B28] Hu X. X. Chi H. L. Fu X. Y. Chen J. L. Dong L. Y. Jiang S. Q. (2024). Tunable multivalent aptamer-based DNA nanostructures to regulate Multiheteroreceptor-Mediated tumor recognition. J. Am. Chem. Soc. 146 (4), 2514–2523. 10.1021/jacs.3c10704 38247135

[B29] Huang M. S. Christakopoulos F. Roth J. G. Heilshorn S. C. (2024). Organoid bioprinting: from cells to functional tissues. Nat. Rev. Bioeng. 3 (2), 126–142. 10.1038/s44222-024-00268-0

[B30] Jaganathan H. Gage J. Leonard F. Srinivasan S. Souza G. R. Dave B. (2014). Three-Dimensional *in vitro* Co-Culture model of breast tumor using magnetic levitation. Sci. Rep. 4, 6468. 10.1038/srep06468 25270048 PMC4180823

[B31] Jin C. He J. X. Zou J. M. Xuan W. J. Fu T. Wang R. W. (2019). Phosphorylated lipid-conjugated oligonucleotide selectively anchors on cell membranes with high alkaline phosphatase expression. Nat. Commun. 10, 2704. 10.1038/s41467-019-10639-6 31221964 PMC6586821

[B32] Jo Y. Choi N. Kim K. Koo H. J. Choi J. Kim H. N. (2018). Chemoresistance of cancer cells: requirements of tumor microenvironment-mimicking *in vitro* models in anti-cancer drug development. Theranostics 8 (19), 5259–5275. 10.7150/thno.29098 30555545 PMC6276092

[B33] Kim J. I. Miura Y. Li M. Y. Revah O. Selvaraj S. Birey F. (2024). Human assembloids reveal the consequences of *CACNA1G* gene variants in the thalamocortical pathway. Neuron 112 (24), 4048–4059.e7. 10.1016/j.neuron.2024.09.020 39419023

[B34] Kim J. I. Imaizumi K. Jurjut O. Kelley K. W. Wang D. Thete M. V. (2025). Human assembloid model of the ascending neural sensory pathway. Nature 642 (8066), 31. 10.1038/s41586-025-08808-3 40205039 PMC12137141

[B35] Kong Y. H. Du Q. Zhao D. Wen Y. J. Zhang T. Q. Geng Z. W. (2025). DNA-programmed responsive microorganism assembly with controlled patterns and behaviors. Sci. Adv. 11 (24), eads8651. 10.1126/sciadv.ads8651 40512851 PMC12164988

[B36] Kwak M. Herrmann A. (2011). Nucleic acid amphiphiles: synthesis and self-assembled nanostructures. Chem. Soc. Rev. 40 (12), 5745–5755. 10.1039/c1cs15138j 21858338

[B37] Lancaster M. A. Knoblich J. A. (2014). Organogenesis in a dish: modeling development and disease using organoid technologies. Science 345 (6194), 1247125. 10.1126/science.1247125 25035496

[B38] Lancaster M. A. Renner M. Martin C. A. Wenzel D. Bicknell L. S. Hurles M. E. (2013). Cerebral organoids model human brain development and microcephaly. Nature 501 (7467), 373–379. 10.1038/nature12517 23995685 PMC3817409

[B39] Lancaster M. A. Corsini N. S. Wolfinger S. Gustafson E. H. Phillips A. W. Burkard T. R. (2017). Guided self-organization and cortical plate formation in human brain organoids. Nat. Biotechnol. 35 (7), 659–666. 10.1038/nbt.3906 28562594 PMC5824977

[B40] Li C. Faulkner-Jones A. Dun A. R. Jin J. Chen P. Xing Y. Z. (2015). Rapid Formation of a supramolecular Polypeptide-DNA hydrogel for *in situ* three-dimensional multilayer bioprinting. Angew. Chem. Int. Ed. 54 (13), 3957–3961. 10.1002/anie.201411383 25656851

[B41] Li X. J. Xie X. D. Ma Z. W. Li Q. Liu L. Hu X. J. (2018). Programming niche accessibility and *in vitro* stemness with intercellular DNA reactions. Adv. Mat. 30 (46), 1804861. 10.1002/adma.201804861 30276898

[B42] Li H. Y. Liu Q. Crielaard B. J. de Vries J. W. Loznik M. Meng Z. J. (2019a). Fast, efficient, and targeted liposome delivery mediated by DNA hybridization. Adv. Healthc. Mat. 8 (14), 1900389. 10.1002/adhm.201900389 31081288

[B43] Li L. Chen X. G. Cui C. Pan X. S. Li X. W. Yazd H. S. (2019b). Aptamer displacement reaction from live-cell surfaces and its applications. J. Am. Chem. Soc. 141 (43), 17174–17179. 10.1021/jacs.9b07191 31539233

[B105] Li J. Xun K. Y. Pei K. Liu X. J. Peng X. Y. Du Y. L. (2019). Cell-membrane-anchored DNA nanoplatform for programming cellular interactions. J. Am. Chem. Soc. 141 (45), 18013–18020. 10.1021/jacs.9b04725 31626550

[B44] Li L. Yin J. Ma W. Tang L. G. Zou J. H. Yang L. Z. (2024). A DNA origami device spatially controls CD95 signalling to induce immune tolerance in rheumatoid arthritis. Nat. Mat. 23 (7), 993–1001. 10.1038/s41563-024-01865-5 38594486

[B45] Li L. X. Liu S. Zhu C. D. Shao S. X. Yang F. Liu Q. L. (2025a). DNA-Based signal circuit for self-regulated bidirectional communication in protocell-living cell communities. Angew. Chem. Int. Ed. 64 (24), e202503903. 10.1002/anie.202503903 40195061

[B46] Li M. Q. Xu Y. P. Li K. Zhou C. Fan X. X. Wang H. (2025b). Recapitulating dengue virus infection with human pluripotent stem cell-derived liver organoids for antiviral screening. Nat. Commun. 16 (1), 8069. 10.1038/s41467-025-63323-3 40877283 PMC12394640

[B47] Li Q. Wang L. L. Xie W. L. Tan Q. Q. Zhou S. Pei H. M. (2025c). Dynamic modulation of interactions between hydrophobic tag-conjugated DNA nanoprobes and cell membrane facilitates ATP imaging in the tumor microenvironment. Angew. Chem. Int. Ed. 64 (25), e202505223. 10.1002/anie.202505223 40230174

[B48] Lin M. J. Chen Y. Y. Zhao S. S. Tang R. Nie Z. Xing H. (2022). A biomimetic approach for spatially controlled cell membrane engineering using Fusogenic spherical nucleic acid. Angew. Chem. Int. Ed. 61 (1), e202111647. 10.1002/anie.202111647 34637590

[B49] Liu J. S. Farlow J. T. Paulson A. K. Labarge M. A. Gartner Z. J. (2012). Programmed cell-to-cell variability in ras activity triggers emergent behaviors during mammary epithelial morphogenesis. Cell. Rep. 2 (5), 1461–1470. 10.1016/j.celrep.2012.08.037 23041312 PMC3634587

[B50] Liu B. Qi Z. Chao J. (2023). Framework nucleic acids directed assembly of Au nanostructures for biomedical applications. Interdiscip. Med. 1 (1), e20220009. 10.1002/inmd.20220009

[B51] Lo H. C. Choi H. Kolahi K. Rodriguez S. Gonzalez L. Chu F. L. (2025). A 3D tumor spheroid model with robust T cell infiltration for evaluating immune cell engagers. iScience 28 (8), 112996. 10.1016/j.isci.2025.112996 40703438 PMC12283745

[B52] Louis B. Tewary M. Bremer A. W. Philippeos C. Negri V. A. Zijl S. (2022). A reductionist approach to determine the effect of cell-cell contact on human epidermal stem cell differentiation. Acta Biomater. 150, 265–276. 10.1016/j.actbio.2022.07.054 35926780 PMC9810539

[B53] Luo Y. Y. Hao Y. R. Sun C. Y. Lu Z. Wang H. Lin Y. H. (2025). Gut-derived indole propionic acid alleviates liver fibrosis by targeting profibrogenic macrophages *via* the gut-liver axis. Cell. Mol. Immunol. 13. 10.1038/s41423-025-01339-x 40926017 PMC12575719

[B54] Lv Q. Zhao X. Teng S. S. Jin X. M. Zhou Y. Sun Y. Y. (2025). DNA origami-based CD44-Targeted therapy silences Stat3 enhances cartilage regeneration and alleviates osteoarthritis progression. Adv. Sci. 12 (29), e03939. 10.1002/advs.202503939 40396977 PMC12362795

[B55] Ma Y. H. Zhu Y. Wu H. He Y. Zhang Q. Huang Q. L. (2024). Domain-Targeted membrane partitioning of specific proteins with DNA nanodevices. J. Am. Chem. Soc. 146 (11), 7640–7648. 10.1021/jacs.3c13966 38466380

[B56] Martinez N. J. Titus S. A. Wagner A. K. Simeonov A. (2015). High-throughput fluorescence imaging approaches for drug discovery using *in vitro* and *in vivo* three-dimensional models. Expert.Opin.Drug Discov. 10 (12), 1347–1361. 10.1517/17460441.2015.1091814 26394277 PMC4863443

[B57] Mathis K. Kohon A. I. Black S. Meckes B. (2023). Light-Controlled cell-cell assembly using photocaged oligonucleotides. ACS Mater. Au 3 (4), 386–393. 10.1021/acsmaterialsau.3c00020 38090125 PMC10347689

[B58] Mathis K. Chan C. T. Y. Meckes B. (2024). Controlling cell interactions with DNA directed assembly. Adv. Healthc. Mat. 13 (32), 2402876. 10.1002/adhm.202402876 39402803 PMC11671287

[B59] Ni S. J. Yao H. Z. Wang L. L. Lu J. Jiang F. Lu A. P. (2017). Chemical modifications of nucleic acid aptamers for therapeutic purposes. Int. J. Mol. Sci. 18 (8), 1683. 10.3390/ijms18081683 28767098 PMC5578073

[B60] Park S. E. Georgescu A. Huh D. (2019). Organoids-on-a-chip. Science 364 (6444), 960–965. 10.1126/science.aaw7894 31171693 PMC7764943

[B61] Pasca S. P. Arlotta P. Bateup H. S. Camp J. G. Cappello S. Gage F. H. (2025). A framework for neural organoids, assembloids and transplantation studies. Nature 639 (8054), 315–320. 10.1038/s41586-024-08487-6 39653126

[B62] Peng Y. H. Hsiao S. K. Gupta K. Ruland A. Auernhammer G. K. Maitz M. F. (2024). Author Correction: dynamic matrices with DNA-encoded viscoelasticity for cell and organoid culture. Nat. Nanotechnol. 19 (3), 418. 10.1038/s41565-024-01619-z 38297147 PMC10950782

[B63] Porter C. M. Qian G. C. Grindel S. H. Hughes A. J. (2024). Highly parallel production of designer organoids by mosaic patterning of progenitors. Cell. Syst. 15 (7), 649–661.e9. 10.1016/j.cels.2024.06.004 38981488 PMC11257788

[B64] Prahl L. S. Porter C. M. Liu J. G. Viola J. M. Hughes A. J. (2023). Independent control over cell patterning and adhesion on hydrogel substrates for tissue interface mechanobiology. iScience 26 (1), 106657. 10.1016/j.isci.2023.106657 37168559 PMC10164898

[B65] Qian R. C. Zhou Z. R. Guo W. J. Wu Y. T. Yang Z. L. Lu Y. (2021). Cell surface engineering using DNAzymes: metal ion mediated control of cell-cell interactions. J. Am. Chem. Soc. 143 (15), 5737–5744. 10.1021/jacs.1c00060 33749281 PMC9170360

[B66] Rookmaaker M. B. Schutgens F. Verhaar M. C. Clevers H. (2015). Development and application of human adult stem or progenitor cell organoids. Nat. Rev. Nephrol. 11 (9), 546–554. 10.1038/nrneph.2015.118 26215513

[B67] Rothemund P. W. K. (2006). Folding DNA to create nanoscale shapes and patterns. Nature 440 (7082), 297–302. 10.1038/nature04586 16541064

[B68] Saka S. K. Wang Y. Kishi J. Y. Zhu A. Zeng Y. T. Xie W. X. (2019). Immuno-SABER enables highly multiplexed and amplified protein imaging in tissues. Nat. Biotechnol. 37 (9), 1080–1090. 10.1038/s41587-019-0207-y 31427819 PMC6728175

[B69] Scheideler O. J. Yang C. Kozminsky M. Mosher K. I. Falcón-Banchs R. Ciminelli E. C. (2020). Recapitulating complex biological signaling environments using a multiplexed, DNA-patterning approach. Sci. Adv. 6 (12), eaay5696. 10.1126/sciadv.aay5696 32206713 PMC7080440

[B70] Selden N. S. Todhunter M. E. Jee N. Y. Liu J. S. Broaders K. E. Gartner Z. J. (2012). Chemically programmed cell adhesion with membrane-anchored oligonucleotides. J. Am. Chem. Soc. 134 (2), 765–768. 10.1021/ja2080949 22176556 PMC3280587

[B71] Shen C. Y. Wang J. Li G. F. Hao S. Y. Wu Y. Song P. R. (2024). Boosting cartilage repair with silk fibroin-DNA hydrogel-based cartilage organoid precursor. Bioact. Mat. 35, 429–444. 10.1016/j.bioactmat.2024.02.016 38390528 PMC10881360

[B72] Shi P. Wang Y. (2021). Synthetic DNA for cell-surface engineering. Angew. Chem. Int. Ed. 60 (21), 11580–11591. 10.1002/anie.202010278 33006229

[B73] Shi P. Zhao N. Lai J. P. Coyne J. Gaddes E. R. Wang Y. (2018). Polyvalent display of biomolecules on live cells. Angew. Chem. Int. Ed. 57 (23), 6800–6804. 10.1002/anie.201712596 29380466 PMC5976537

[B74] Stephan M. T. Moon J. J. Um S. H. Bershteyn A. Irvine D. J. (2010). Therapeutic cell engineering with surface-conjugated synthetic nanoparticles. Nat. Med. 16 (9), 1035–1041. 10.1038/nm.2198 20711198 PMC2935928

[B75] Stevens A. J. Harris A. R. Gerdts J. Kim K. H. Trentesaux C. Ramirez J. T. (2023). Programming multicellular assembly with synthetic cell adhesion molecules. Nature 614 (7946), 144–152. 10.1038/s41586-022-05622-z 36509107 PMC9892004

[B76] Sun L. L. Gao Y. J. Wang Y. G. Wei Q. Shi J. Y. Chen N. (2018). Guiding protein delivery into live cells using DNA-programmed membrane fusion. Chem. Sci. 9 (27), 5967–5975. 10.1039/c8sc00367j 30079211 PMC6050539

[B77] Todhunter M. E. Jee N. Y. Hughes A. J. Coyle M. C. Cerchiari A. Farlow J. (2015). Programmed synthesis of three-dimensional tissues. Nat. Methods. 12 (10), 975–981. 10.1038/nmeth.3553 26322836 PMC4589502

[B78] Um S. H. Lee J. B. Park N. Kwon S. Y. Umbach C. C. Luo D. (2006). Enzyme-catalysed assembly of DNA hydrogel. Nat. Mat. 5 (10), 797–801. 10.1038/nmat1741 16998469

[B79] Vermesh U. Vermesh O. Wang J. Kwong G. A. Ma C. Hwang K. (2011). High-Density, multiplexed patterning of cells at single-cell resolution for tissue engineering and other applications. Angew. Chem. Int. Ed. 50 (32), 7378–7380. 10.1002/anie.201102249 21717543 PMC3651859

[B80] Viola J. M. Porter C. M. Gupta A. Alibekova M. Prahl L. S. Hughes A. J. (2020). Guiding cell network assembly using shape-morphing hydrogels. Adv. Mat. 32 (31), 2002195. 10.1002/adma.202002195 32578300 PMC7950730

[B81] Vogel K. Glettenberg M. Schroeder H. Niemeyer C. M. (2013). DNA-Modification of eukaryotic cells. Small 9 (2), 255–262. 10.1002/smll.201201852 23109119

[B82] Wagenbauer K. F. Pham N. Gottschlich A. Kick B. Kozina V. Frank C. (2023). Programmable multispecific DNA-origami-based T-cell engagers. Nat. Nanotechnol. 18 (11), 1319–1326. 10.1038/s41565-023-01471-7 37591933 PMC10656288

[B83] Wang S. H. Wang X. M. Boone J. Wie J. Yip K. P. Zhang J. (2017a). Application of hanging drop technique for kidney tissue culture. Kidney Blood Press.Res 42 (2), 220–231. 10.1159/000476018 28478441 PMC6050513

[B84] Wang Y. J. Shao Y. Ma X. Z. Zhou B. N. Faulkner-Jones A. Shu W. M. (2017b). Constructing tissuelike complex structures using cell-laden DNA hydrogel bricks. ACS Appl.Mater.Interfaces 9 (14), 12311–12315. 10.1021/acsami.7b01604 28300395

[B85] Wang K. Z. Wei Y. H. Xie X. D. Li Q. Liu X. G. Wang L. H. (2023). DNA-Programmed stem cell niches *via* orthogonal extracellular vesicle-cell communications. Adv. Mat. 35 (45), 2302323. 10.1002/adma.202302323 37463346

[B86] Wang R. B. Liu Y. H. Zhang Y. L. Yi Q. X. Xiao W. J. Wang T. Q. (2025). DNA framework-enabled ocular barrier penetration for microinvasive antiangiogenic therapy. J. Am. Chem. Soc. 147 (9), 7545–7554. 10.1021/jacs.4c16529 39979822

[B87] Weber R. J. Cerchiari A. E. Delannoy L. S. Garbe J. C. LaBarge M. A. Desai T. A. (2016). Rapid organoid reconstitution by chemical micromolding. ACS Biomater.Sci.Eng. 2 (11), 1851–1855. 10.1021/acsbiomaterials.6b00421 33440521

[B88] Wei J. Y. Sun Y. Y. Wang H. M. Zhu T. Li L. Zhou Y. (2024). Designer cellular spheroids with DNA origami for drug screening. Sci. Adv. 10 (29), eado9880. 10.1126/sciadv.ado9880 39028810 PMC11259176

[B89] Wen N. C. Lu Y. Zhuo Y. T. Fu B. Wang H. Y. He Y. (2025). Enhancing T-Cell infiltration and immunity in solid tumors *via* DNA nanolinker-mediated Monocyte hitchhiking. J. Am. Chem. Soc. 147 (11), 9800–9809. 10.1021/jacs.4c18455 40042588

[B90] Xia X. Shi B. Wang L. Liu Y. Zou Y. Zhou Y. (2021). From mouse to mouse-ear cress: nanomaterials as vehicles in plant biotechnology. Explor. (Beijing) 1 (1), 9–20. 10.1002/EXP.20210002 37366467 PMC10291572

[B91] Xiao M. S. Lai W. Yao X. W. Pei H. Fan C. H. Li L. (2022). Programming receptor clustering with DNA probabilistic circuits for enhanced natural killer cell Recognition. Angew. Chem. Int. Ed. 61 (28), e202203800. 10.1002/anie.202203800 35523723

[B92] Xing Y. Z. Cheng E. J. Yang Y. Chen P. Zhang T. Sun Y. W. (2011). Self-Assembled DNA hydrogels with designable thermal and enzymatic responsiveness. Adv. Mat. 23 (9), 1117–1121. 10.1002/adma.201003343 21181766

[B93] Xu W. W. Wang Q. T. Tang C. Y. Qiao Y. L. Feng L. Song D. J. (2025a). Intelligent DNA nanodevice for accurate modulation of cellular behaviors and intercellular interactions *in vitro* . ACS Appl.Mater.Interfaces 17 (26), 38669–38677. 10.1021/acsami.5c05813 40536440

[B94] Xu Y. Luo Y. Lu X. Y. Ye J. Y. Chen Z. Y. Hu Y. (2025b). Dynamic modulation of multicellular interactions *via* ATP-Dissipative DNA assembly. J. Am. Chem. Soc. 147, 28277–28288. 10.1021/jacs.5c08925 40699155

[B96] Yamada T. Trentesaux C. Brunger J. M. Xiao Y. N. Stevens A. J. Martyn I. (2025). Synthetic organizer cells guide development *via* spatial and biochemical instructions. Cell. 188 (3), 778–795.e18. 10.1016/j.cell.2024.11.017 39706189 PMC12027307

[B97] Yang Y. R. Liu Y. Yan H. (2015). DNA nanostructures as programmable biomolecular scaffolds. Bioconjugate Chem. 26 (8), 1381–1395. 10.1021/acs.bioconjchem.5b00194 25961418

[B98] Yao X. X. He D. D. Wei P. Y. Niu Z. T. Chen H. Li L. (2024). DNA nanomaterial-empowered surface engineering of extracellular vesicles. Adv. Mat. 36 (37), 2306852. 10.1002/adma.202306852 38041689

[B99] You M. X. Lyu Y. F. Han D. Qiu L. P. Liu Q. L. Chen T. (2017). DNA probes for monitoring dynamic and transient molecular encounters on live cell membranes. Nat. Nanotechnol. 12 (5), 453–459. 10.1038/nnano.2017.23 28319616 PMC5507702

[B100] Yu H. X. Alkhamis O. Canoura J. Liu Y. Z. Xiao Y. (2021). Advances and challenges in small-molecule DNA aptamer isolation, characterization, and sensor development. Angew. Chem. Int. Ed. 60 (31), 16800–16823. 10.1002/anie.202008663 33559947 PMC8292151

[B101] Zaret K. S. (2002). Regulatory phases of early liver development: paradigms of organogenesis. Nat. Rev. Genet. 3 (7), 499–512. 10.1038/nrg837 12094228

[B102] Zhang Q. Zhang Y. Wu L. M. Wang D. Zhuo Y. T. Lu Y. (2024). DNA reaction circuits to establish designated biological functions in multicellular community. Nano Lett. 24 (19), 5808–5815. 10.1021/acs.nanolett.4c00980 38710049

[B103] Zhu Q. Y. He X. F. Liu J. H. Wang H. M. Shan X. J. Song G. Q. (2024). DNA origami assembled spheroid for evaluating cytotoxicity and infiltration of chimeric antigen receptor macrophage (CAR-M). Commun. Biol. 7 (1), 1302. 10.1038/s42003-024-07009-4 39390143 PMC11467189

[B104] Zhu M. R. Zhang H. Zhou Q. R. Sheng S. H. Gao Q. M. Geng Z. (2025). Dynamic GelMA/DNA dual-network hydrogels promote woven bone organoid Formation and enhance bone regeneration. Adv. Mat. 37 (24), 2501254. 10.1002/adma.202501254 40123197

